# The cleavage of indinavir sulfate: synthesis and characterization of a *cis*-1-amino-2-indanol salt

**DOI:** 10.1107/S2053229625005807

**Published:** 2025-08-05

**Authors:** Tebogo M. L. Mokoto, Andreas Lemmerer, Yasien Sayed, Kgaugelo C. Tapala, Itumeleng B. Setshedi, Mark G. Smith

**Affiliations:** a Protein Structure-Function Research Unit, School of Molecular and Cell Biology, University of the Witwatersrand, Johannesburg, Gauteng, South Africa; b University of South Africa, Department of Chemistry, Unisa Science Campus, Roodepoort, Gauteng, South Africa; c Molecular Sciences Institute, School of Chemistry, University of the Witwatersrand, Johannesburg, Gauteng, South Africa; d University of South Africa, Department of Life and Consumer Sciences, Unisa Science Campus, Roodepoort, Gauteng, South Africa; Universidade Federal de Minas Gerais, Brazil

**Keywords:** crystal structure, indinavir, solvation crystallization, salt crystal structure, slow evaporation, thermal analysis, surface analysis, chemical transformations

## Abstract

The HIV-1 protease inhibitor indinavir sulfate was cleaved *via* a one-pot reflux synthesis using 1-propanol, yielding the salt bis(2-hydroxy-2,3-dihydro-1*H*-inden-1-aminium) sulfate, 2C_9_H_12_NO^+^·SO_4_^2−^. Single-crystal X-ray diffraction (SC-XRD) revealed that the salt crystallizes in the monoclinic space group *P*2_1_. The structure consists of two conformationally distinct cations and one sulfate anion, stabilized through an extensive hydrogen-bonding network. Thermal analysis showed minor solvent loss around 200 °C, followed by a two-step decomposition process commencing at 306.6 °C. Hirshfeld surface analysis revealed dominant O⋯H/H⋯O (44.4–41.0%) and H⋯H (45.2–40.1%) intermolecular contacts, with minor contributions from C⋯H/H⋯C and C⋯O/O⋯C interactions. These contact percentages were calculated for each of the two independent cations. The van der Waals surface area (687.30 Å^2^) accounts for 71.43% of the unit cell. These results provide structural and thermal evidence for the transformation of indinavir sulfate under alcoholytic conditions, highlighting the formation and stabilization of the resulting salt.

## Abstract 2

The cleavage of the HIV-1 protease inhibitor indinavir sulfate *via* a one-pot synthesis reflux method with 1-propanol successfully yielded the salt bis­(2-hy­droxy-2,3-di­hydro-1*H*-inden-1-aminium) sulfate. The reported analysis provides com­prehensive insights into the chemical transformations, thermal stability and mol­ecular inter­actions of the salt, contributing to its characterization and potential pharmaceutical applications.

## Introduction

Amino alcohols are primarily derived from a diverse range of chiral com­pounds, offering significant advantages due to their conformational properties and structural diversity in drug design (Matamoros *et al.*, 2023 ▸). One notable com­pound in this category is *cis*-1-amino-2-indanol, a versatile building block in pharmaceutical formulations. Its applications extend to the synthesis of anti­malarial drugs and HIV-1 protease inhibitors, where it plays a critical role. This com­pound is frequently used as a rigid scaffold for covalently attached chiral auxiliary mol­ecules, acts as a resolving agent for secondary alcohols and can function as a racemic carb­oxy­lic acid. Furthermore, it is instrumental in the catalytic enanti­oselective synthesis of various mol­ecules (Gallou & Senanayake, 2006 ▸). Among amino alcohols, *cis*-1-amino-2-indanol is of considerable phar­ma­ceuti­cal significance (Gallou & Senanayake, 2006 ▸). It was first introduced as a chiral amino alcohol P2′ ligand in the development of HIV protease inhibitors, culminating in Merck’s discovery of the potent inhibitor L-685,434, com­monly known as indinavir (Dorsey *et al.*, 1994 ▸). This chiral com­pound has been recognised as a pivotal ligand in the development of HIV-PR inhibitors, with various strategies employed to synthesize dif­ferent asymmetric constrained phenyl glycinol surrogates. Its sterically bulky indane structure, combined with the restricted *cis*-amino­indanol moiety, constrains the formation of an effective chiral discriminative environment, limiting enanti­oselective or diastereoselective outcomes during reactions (Gallou & Senanayake, 2006 ▸).

Drug hydrolysis plays a crucial role in pharmaceutical research, as it directly affects the stability, efficacy and safety of drug formulations (Mehta & Bhayani, 2017 ▸). This chemical process, which involves breaking down drug mol­ecules through a reaction with water, is a common pathway for drug degradation (Lieberman & Vemuri, 2015 ▸). In addition to water, solvents such as alcohols and other organic solvents can also influence drug hydrolysis (Mehta & Bhayani, 2017 ▸). These solvents may act as reactants or catalysts (Dutta *et al.*, 2024 ▸) in hydrolytic reactions, depending on the chemical nature of the drug and the solvent environment. Hydrolysis can affect various functional groups in drug mol­ecules, such as esters, amides and lactones, leading to therapeutic effectiveness and potential toxicity changes (Zhou *et al.*, 2017 ▸). In certain instances, alcohols can engage in transesterification reactions, where an ester group in the drug mol­ecule inter­acts with the alcohol to create a new ester (Lieberman & Vemuri, 2015 ▸). This reaction is particularly relevant in formulations that include ethanol or other alcohols as solvents. Solvents like acetone, dimethyl sulfoxide (DMSO) or aceto­nitrile can alter the reaction kinetics of hydrolysis. They may stabilize or destabilize inter­mediates, thereby impacting the rate of hydrolysis (Schmidt & Scholze, 1985 ▸; Issa & Luyt, 2019 ▸). The presence of both water and organic solvents can create unique environments that affect hydrolysis.

In recent years, advances in various analytical techniques, such as high-performance liquid chromatography (HPLC), have enhanced our ability to study hydrolytic degradation and predict its impact on drug stability (Battu & Pottabathini, 2015 ▸). Researchers are also exploring innovative formulation strategies, including the use of excipients and encapsulation methods, to mitigate hydrolysis and extend the shelf life of pharmaceutical products. Understanding the mechanisms and factors influencing hydrolysis remains a critical area of focus, as it informs the development of more stable and effective drug formulations.
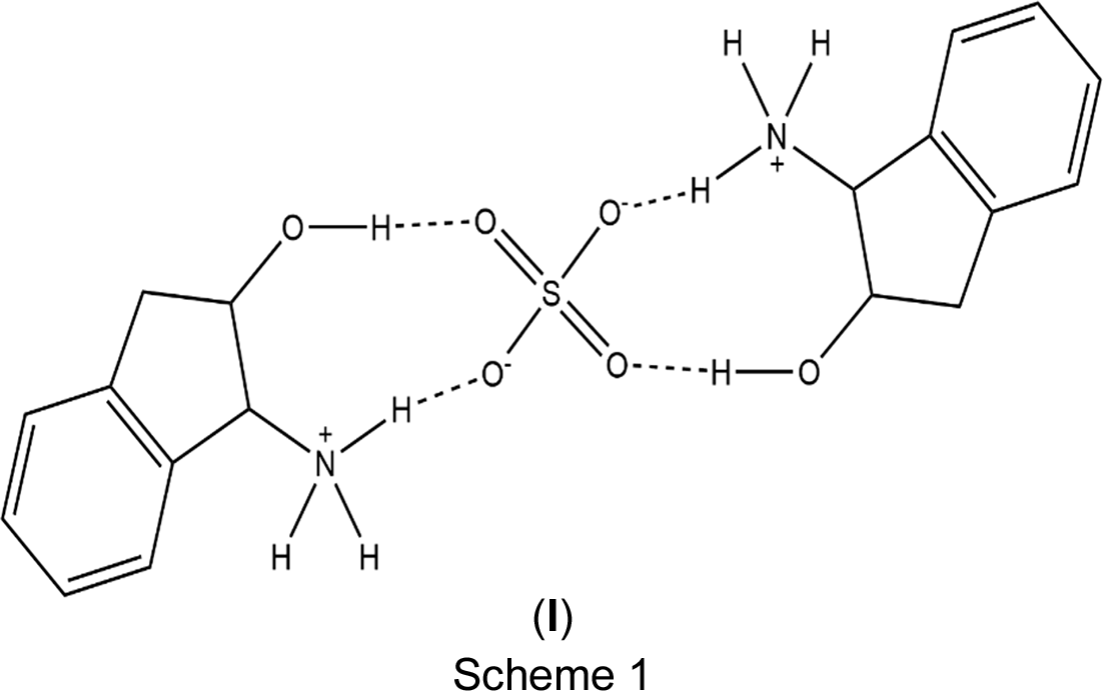


## Experimental

### Synthesis and crystallization

The one-pot synthesis reflux method (Smith *et al.*, 2015 ▸) was used for the synthesis of bis­(2-hy­droxy-2,3-di­hydro-1*H*-inden-1-aminium) sulfate, (**I**) (Scheme 1). In a dram vial, 0.020 g of indinavir sulfate (0.0324 mmol) was dissolved in 1-propanol solvent and heated at 363 K while stirring at 300 rpm for 4 h. With the lid of the dram vial loosely open, the solution was allowed to evaporate slowly at tem­per­a­tures ranging from 283 to 273 K. After three weeks, colourless plates had formed.

### Refinement

Crystal data, data collection and structure refinement details are summarized in Table 1 ▸. H atoms on O and N atoms were allowed to refine freely.

### Surface analyses

Hirshfeld surface analysis was performed to qu­antify the various inter­molecular inter­actions contributing to the crystal structure of salt (**I**) using *CrystalExplorer* (Version 17.5; Spackman *et al.*, 2021 ▸). A surface was mapped over *d*_norm_, along with two-dimensional (2D) fingerprint plots, which were utilized to analyse these inter­actions (McKinnon *et al.*, 2007 ▸; Spackman & Jayatilaka, 2009 ▸). The Hirshfeld surface of salt (**I**) is colour-coded and mapped over *d*_norm_. The surface area, density and volumetric analyses were conducted using the two-probe mode configuration, employing a small radius probe (*r*_small_p_) of 1.2 Å and a large radius probe (*r*_large_p_) of 3.0 Å. Optimization was performed to a depth of 4, with a grid resolution of 0.2 Å for enhanced accuracy using *MoloVol* (Version 1.1.1; Maglic & Lavendomme, 2022 ▸).

### Fourier transform IR (FT–IR) spectroscopy and Raman spectroscopy

FT–IR analysis was performed at 288 K using a Shimadzu QATR10 ATR accessory with a germanium crystal. Measurements were taken in percentage transmittance mode with 64 scans, a resolution of 4 cm^−1^ and a range of 700–4.000 cm^−1^. Data were processed with *LabSolutions IR* (Version 2.26). Raman spectroscopy utilized a Bruker MultiRam Fourier transform spectrometer with a Nd-YAG laser and a germanium diode detector, also with 64 scans at 450 mW power.

### Thermogravimetric analysis (TGA) and dif­ferential scanning calorimetry (DSC)

Thermogravimetric analysis and dif­ferential scanning calorimetry data were collected using a thermogravimetric analyser from an Advanced Laboratory Solutions (TGA/DSC) SQ600 between 323 and 1073 K, at a heating rate of 283 K min^−1^ and a nitro­gen gas flow rate of 20 ml min^−1^.

### Nuclear magnetic resonance (NMR)

For the NMR analysis, crystals of salt (**I**) were dissolved in deuterated DMSO-*d*_6_ containing 1% tetra­methyl­silane and transferred to an NMR tube for analysis. The samples were analysed using an Agilent 500 MHz spectrometer at room tem­per­a­ture (299 K) and the NMR spectra were captured (^1^H at 500 MHz and ^13^C at 125 MHz).

^1^H NMR (500 MHz, DMSO-*d*_6_): δ (ppm) 8.50 (*s*, 1H, –NH_3_), 8.15 (*s*, 1H, –NH_3_), 7.71 (*s*, 1H, –NH_3_), 7.48–7.23 (*m*, 4H, –CH aromatic of the indenol moiety), 5.85 (*s*, 1H, OH), 4.57 [*d*, 1H, –CH(OH)], 3.02–2.98 (*m*, 1H, –CH_2_), 2.95–2.91 (*m*, 1H, –CH_2_). ^13^C NMR (125 MHz, DMSO-*d*_6_): δ (ppm) 141.40 (1C), 138.62 (1C), 129.13 (1C), 128.85 (2C), 126.77 (1C), 70.17 (1C), 62.44 (1C), 35.53 (1C). HRMS–ESI (aceto­nitrile, *m*/*z*) calculated for [C_9_H_12_NO]^+^ 150.09; found: [*M* + 2]^+^ 152.2306.

## Results and discussion

Indinavir sulfate was reacted with 1-propanol, which resulted in the alcoholysis of the salt into two distinct parts. Under certain stress conditions, such as changes in pH, tem­per­a­ture and oxidation, indinavir sulfate is known to hydrolyse and ultimately degrade (Rao *et al.*, 2013 ▸), as observed in the cleavage of the mol­ecule. However, since 1-propanol is an alcohol, the degradation of indinavir sulfate in this case is referred to as alcoholysis. A reaction where an alcohol mol­ecule acts as a reactant, breaking chemical bonds, typically in esters or similar com­pounds, by replacing certain functional groups with an alcohol group (Avhad & Marchetti, 2015 ▸). The com­pound cleaved into (3*S*,5*S*)-3-benzyl-5-[(2-{[(*tert*-butyl­am­ino)­oxy]meth­yl}-4-(pyridin-3-ylmeth­yl)piperazin-1-yl)meth­yl]di­hydro­furan-2(3*H*)-one, which remained in solution, and the salt bis­(2-hy­droxy-2,3-di­hydro-1*H*-inden-1-aminium) sulfate, (**I**) (Scheme 1), which was crystallized.

**Figure 1:**
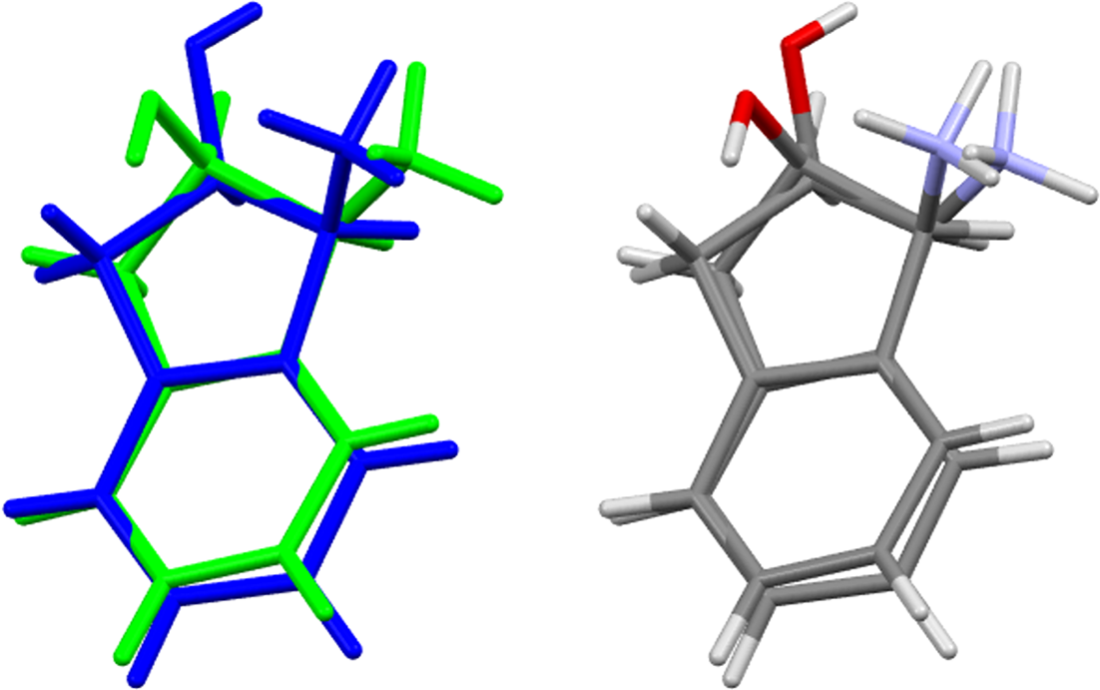
Mol­ecular overlay of the 2-hy­droxy-2,3-di­hydro-1*H*-inden-1-aminium cations, highlighting their minor conformational diversity.

Salt (**I**) crystallized in the monoclinic space group *P*2_1_ with *Z* = 2. The proposed chemical formula for the asymmetric unit is 2C_9_H_12_NO^+^·SO_4_^2−^. The cations are, however, slightly dif­ferent from one another due to the absence of a centre of inversion, indicating that the cations themselves are not symmetrical. The cations exhibit minor conformational diversity. The hy­droxy H atoms point in opposite directions, at an angle of 138° from one another (Fig. 1 ▸).

**Figure 2:**
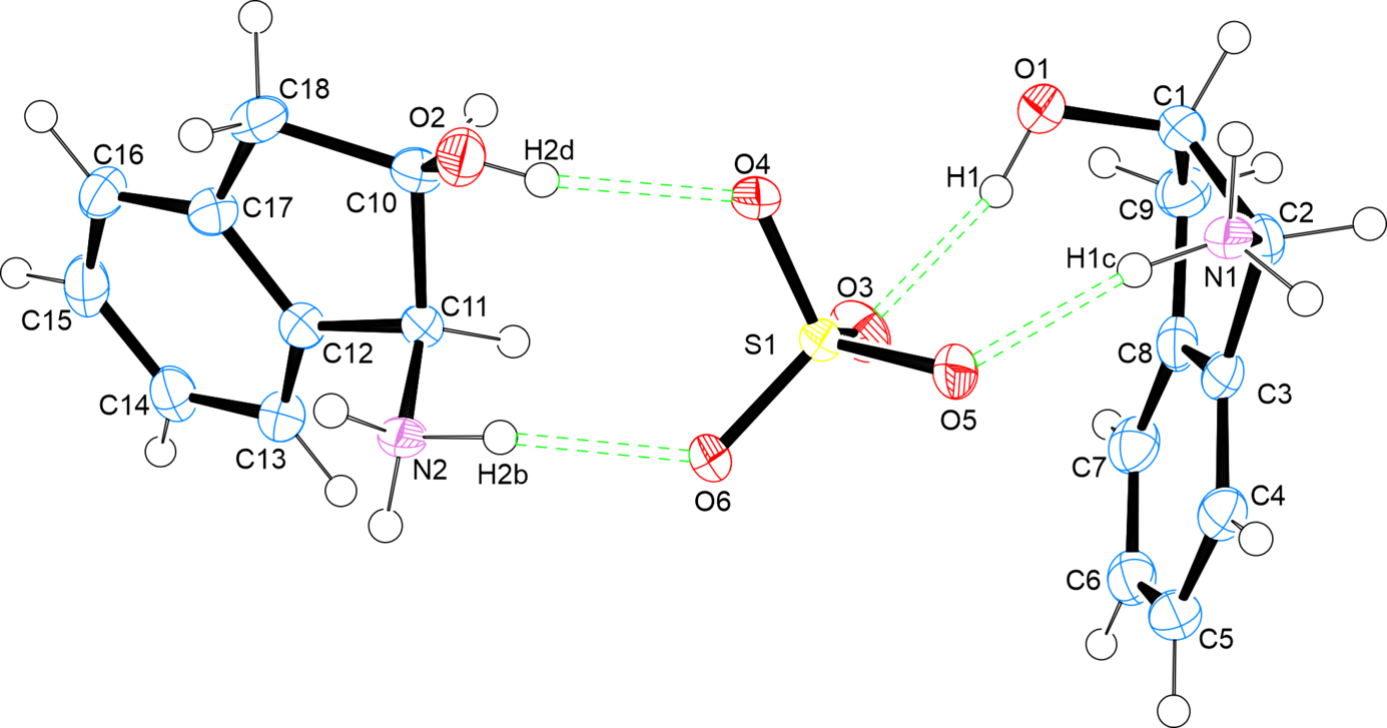
The asymmetric unit of salt (**I**). Displacement ellipsoids are drawn at the 50% probability level.

The asymmetric unit of salt (**I**) is illustrated in Fig. 2 ▸. The figure illustrates that the salt is stabilized by hy­dro­gen bonds, with donor–acceptor distances ranging from 2.6 to 2.9 Å. Both amine functional groups of the cations inter­act with the S=O bonds: N1⋯O5 exhibits a hy­dro­gen-bond length of 2.807 (4) Å, while N2⋯O6 displays a length of 2.833 (3) Å.

Additionally, the alcohol functional groups form hy­dro­gen bonds with the S=O moieties. Specifically, O1⋯O3 features a hy­dro­gen-bond length of 2.724 (3) Å and O2⋯O4 forms a hy­dro­gen bond of 2.675 (3) Å. The bond angles observed in these inter­actions are linked to the sulfate anion. The out-of-plane bond angle for C9—C1—O1 is 112.5 (3)°, while the second cation exhibits a corresponding bond angle for C11—C10—O2 of 113.3 (3)°. Positioned between the two cations, the sulfate anion adopts a tetra­hedral structure with an average O—S—O bond angle of 109.42°. This configuration is characteristic of tetra­hedral mol­ecules and reflects the optimized mol­ecular geometry within the crystal lattice. The bond angles for the sulfate anion are detailed in Table 2 ▸.

**Figure 3:**
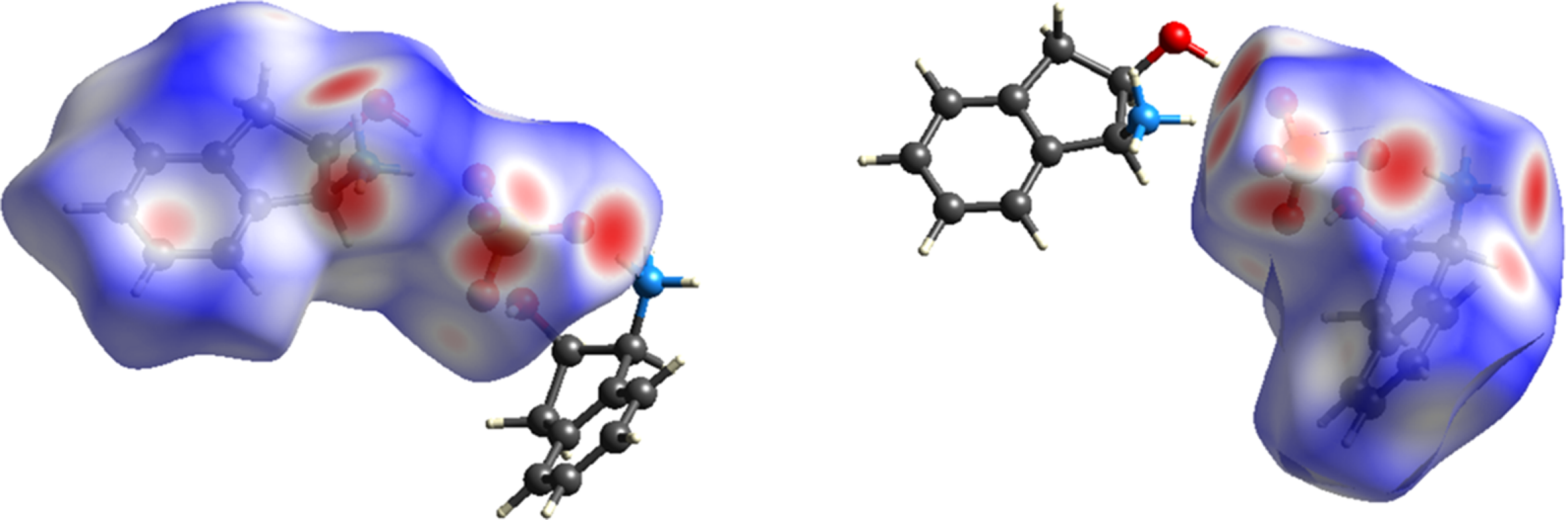
Two views of the independent cation surfaces of the salt (**I**) mapped over *d*_norm_, indicating the various intermolecular bonds in white, red and blue.

Hirshfeld surface analysis was conducted to characterize the intermolecular interactions between each independent cation and the sulfate ion within the asymmetric unit. The molecular assembly is inherently asymmetric, highlighting the noncentrosymmetric nature of the structure. The two-dimensional (2D) fingerprint plots were generated based solely on the asymmetric unit rather than the full packing arrangement within the unit cell (*Z* = 2), ensuring an accurate depiction of the local inter­action motifs rather than global lattice effects (Fig. 3 ▸). The red areas between the aminium and sulfate ions, as well as between the hy­droxy groups and sulfate ions, indicate short inter­molecular bonds, such as strong hy­dro­gen bonding and van der Waals inter­actions, due to the distance between bonds. The blue spots indicate regions in the crystal where the contacts between the atoms are larger than the sum of the van der Waals radii. These contacts are typically weak inter­actions that contribute to less significant inter­molecular bonding.

**Figure 4:**
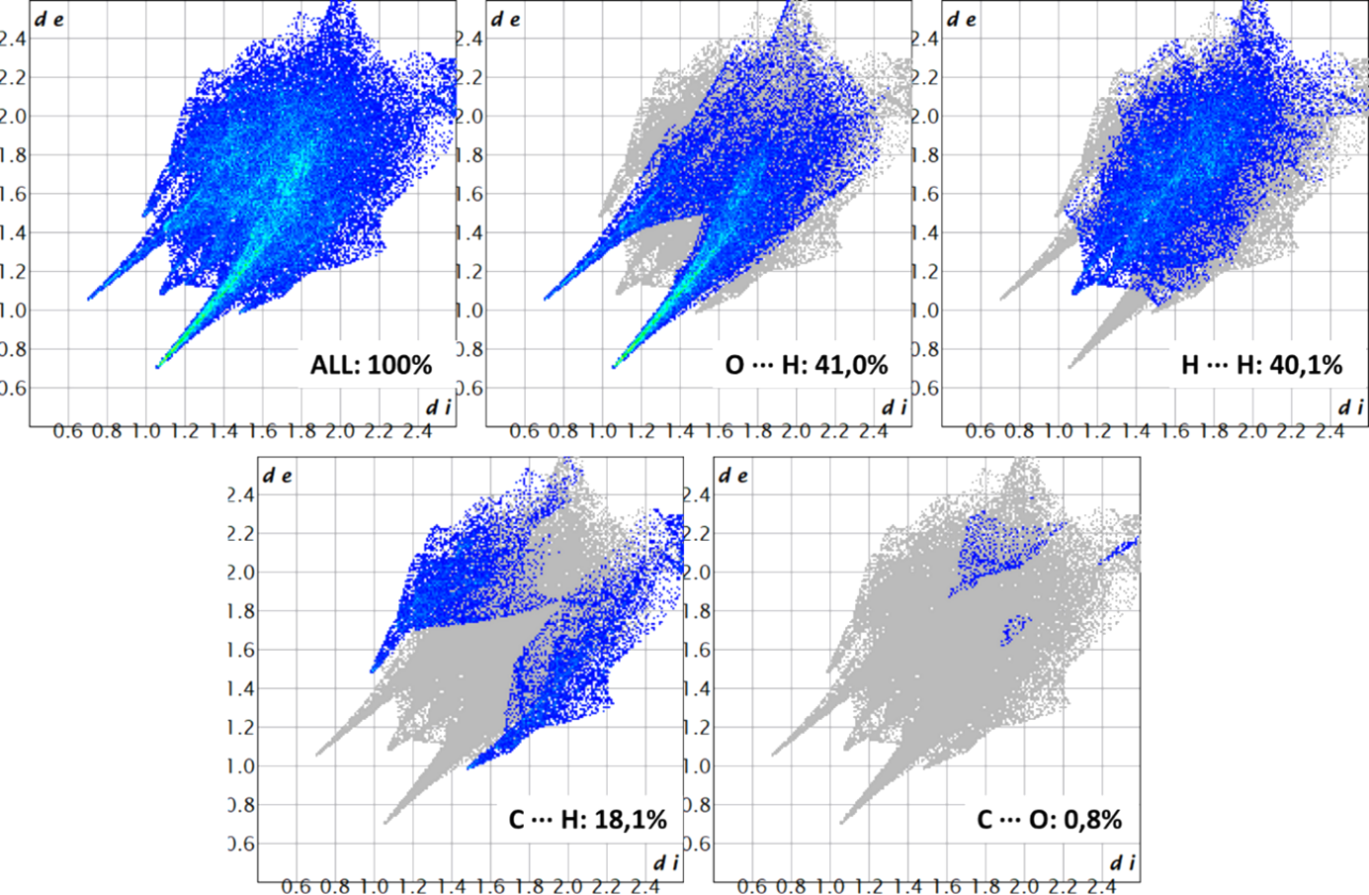
2D fingerprint plots of cation *A* of salt (**I**), detailing the intra­molecular inter­actions.

In the first independent cation (Fig. 3 ▸, left), the dominance of O⋯H/H⋯O interactions at 41.0% aligns with their sig­nifi­cant role in hydrogen bonding, which often drives molecular stability. The closely following H⋯H interactions (40.1%) highlight the contribution of van der Waals forces in stabilizing the system. C⋯H/H⋯C contacts (18.1%) suggest secondary interactions, possibly contributing to the overall packing and orientation of the components. Finally, the minimal C⋯O/O⋯C interactions at 0.8% imply that these interactions play a very limited role in the stability of the system (Fig. 4 ▸).

**Figure 5:**
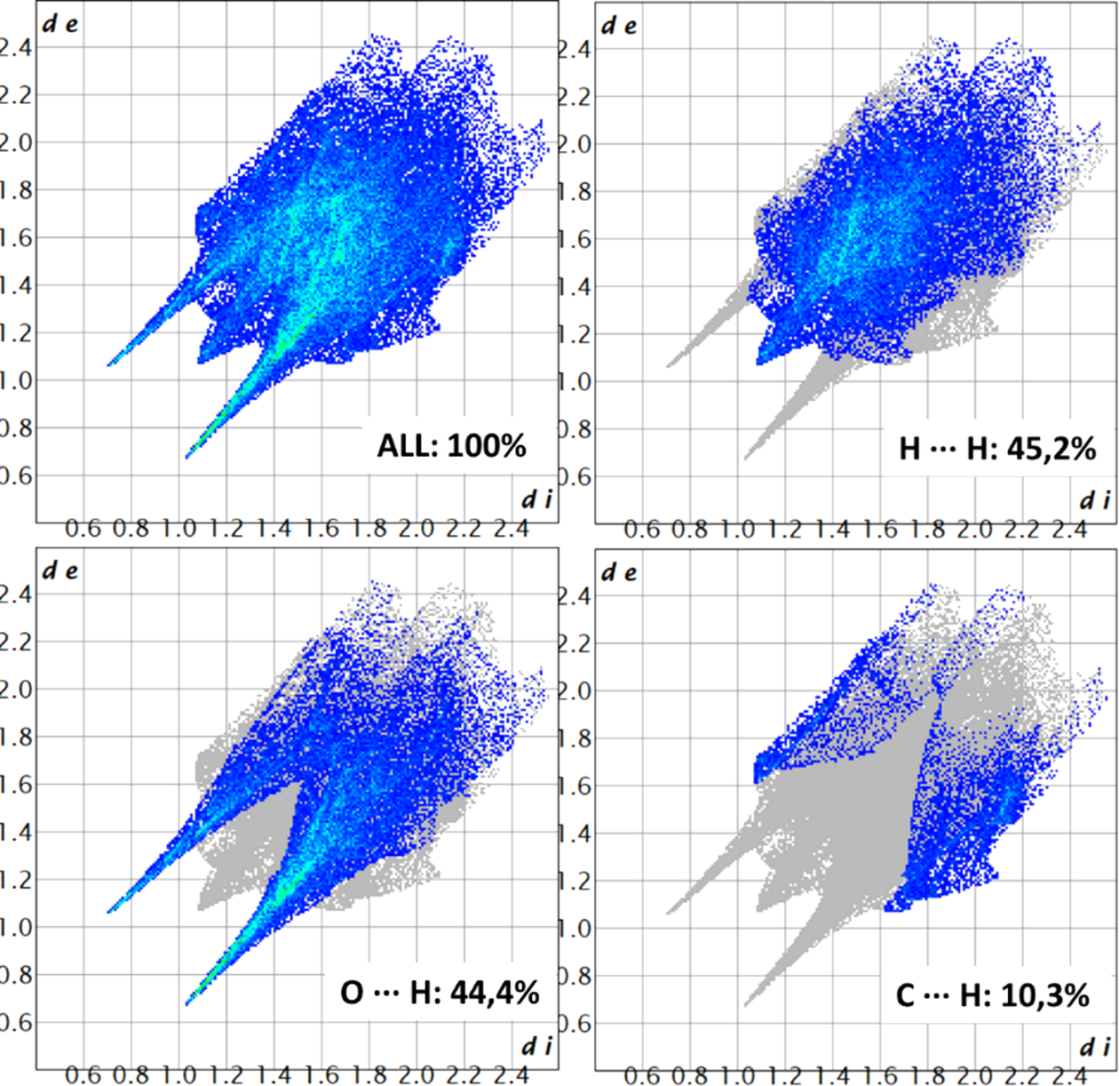
2D fingerprint plots of cation *B* of salt (**I**), detailing the intra­molecular inter­actions.

In the second independent cation (Fig. 3 ▸, right), the interactions within the crystal structure indicate an increase in H⋯H contacts to 45.2%, underscoring their dominant role, likely reflecting stronger van der Waals interactions or a more densely packed molecular environment. The similar percentages of the O⋯H/H⋯O inter­actions at 44.4% highlights the significance of hy­dro­gen bonding, potentially linked to stability or reactivity dif­ferences com­pared to the first part of the structure. The lower proportion of C⋯H/H⋯C inter­actions at 10.3% suggests inter­action variations due to the change in mol­ecular orientation. These changes offer insights into dif­fering behaviour pathways across structural regions. C⋯O inter­actions remain minor, making up just 0.8% here as well (Fig. 5 ▸).

This con­sistency across both parts of the crystal structure suggests that C⋯O contacts have a limited role in stabilizing the mol­ecular framework. However, even such low percentages can occasionally hint at subtle but specific inter­actions, such as dipole–dipole alignments or lone-pair contributions.

**Figure 6:**
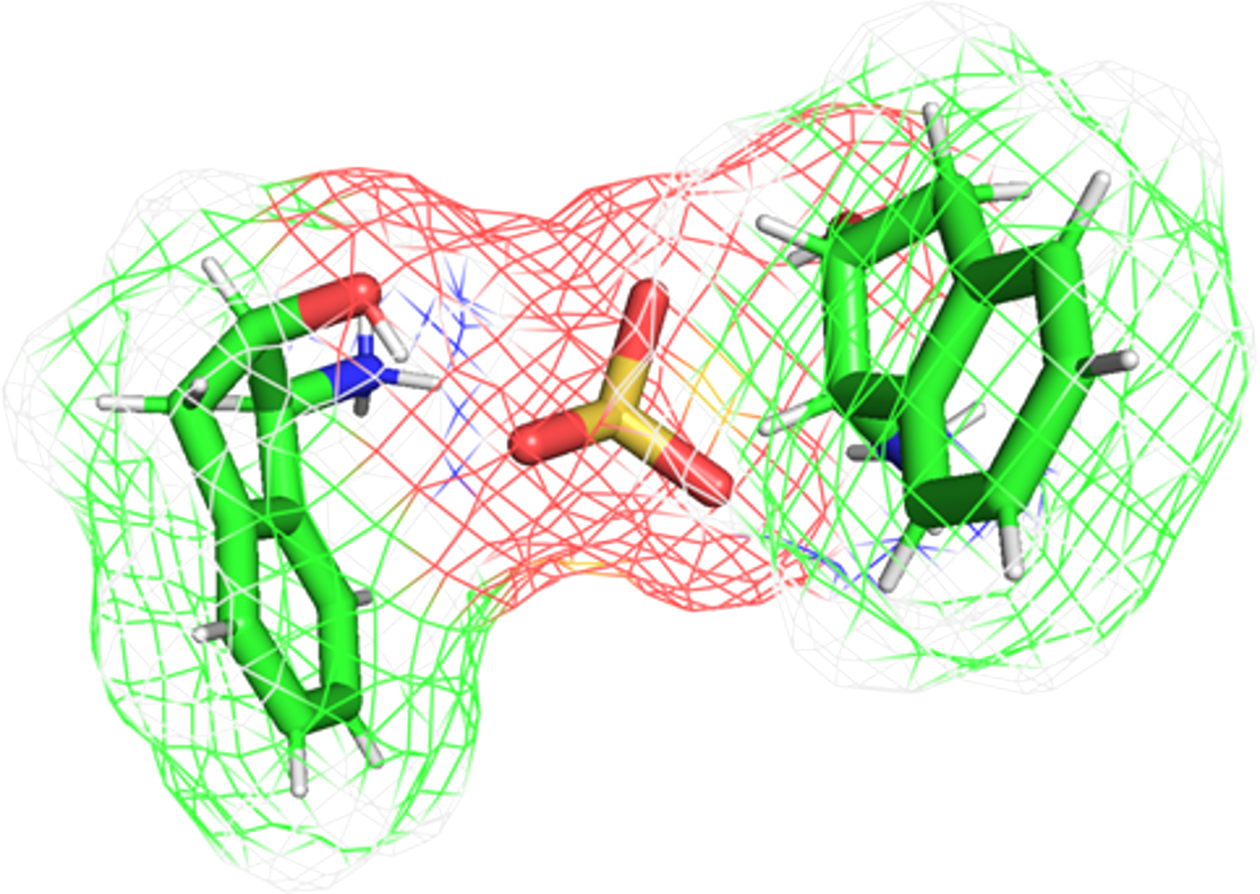
The van der Waals surface area map of the asymmetric unit of salt (**I**).

Further crystallographic details revealed that the calculated density of the crystal is ρ = 1.408 Mg m^−3^. The total mol­ecular volume of the unit cell was measured at 934.95 Å^3^, with van der Waals volumes accounting for 71.43% of the cell, while 28.57% corresponded to probe-excluded void volumes, according to the *MoloVol* volumetric analysis. The calculated van der Waals surface area of the mol­ecule is 687.30 Å^2^ (Fig. 6 ▸) and is solely based on the van der Waals radii of the constituent atoms (Charry & Tkatchenko, 2024 ▸). The calculated van der Waals surface area of 687.30 Å^2^ reflects the size and shape of the molecular envelope over which intermolecular interactions occur. When considered alongside the Hirshfeld surface interaction percentages, this surface area indicates that there is sufficient contact area to support both dense molecular packing, as evidenced by the high proportion of H⋯H contacts, and extensive hydrogen bonding, as seen in the O⋯H/H⋯O contributions. This analysis highlights the spatial distribution of close contacts and underscores the dual role of van der Waals forces and hydrogen bonding in stabilizing the crystal structure.

The degradation product, (1*S*,2*R*)-(+)-*cis*-1-amino-2-in­dan­ol, formed by alcoholysis of the amide bond on indinavir, is enanti­opure and lacks inversion symmetry. This intrinsic chirality drives the selection of space group *P*2_1_, as centrosymmetric packing would require both enanti­omers, which are absent in this case. Furthermore, the sulfate ion plays a crucial role in stabilizing the asym­metric mol­ecular arrangement. The anion engages in specific hy­dro­gen-bonding and electrostatic inter­actions, which reinforce the observed packing mode.

**Figure 7:**
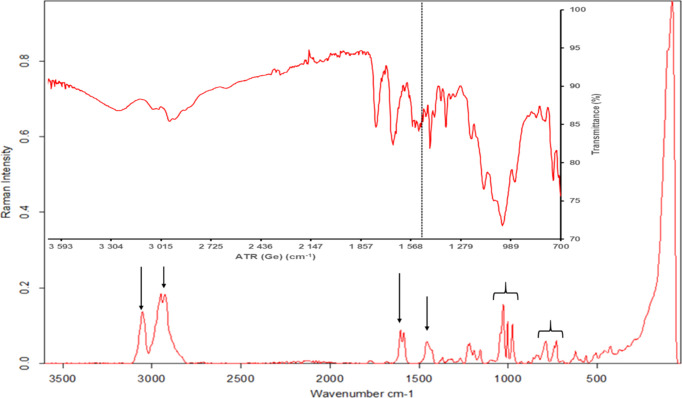
Figure 7: Superimposed FT–IR and Raman spectra of salt (**I**), with arrows indicating major peaks observed in the Raman spectrum.

The FT–IR and Raman spectra of salt (**I**) are depicted in Fig. 7 ▸, and the major peaks are summarized in Table 3 ▸. The strong peak at 2995–2849 cm^−1^ depicts a primary amine peak at 3180–3200 cm^−1^. A 3003–2868 cm^−1^ peak was re­corded, which is observed as a symmetric vibration with another –NH_3_^+^ group at 1650–1580 cm^−1^. The very strong and broad peak at 3100–2400 cm^−1^ indicates the presence of –OH groups. In the range 1060–1045 cm^−1^, there is an S=O stretch, depicted as a very strong peak in the fingerprint region. There is an additional peak that was observed at 1765 cm^−1^, which corresponds with an S=O stretching vibration. The high polarity of S=O leads to a significant change in the dipole moment during the stretching vibration that would appear in the FT–IR spectra. The same distinctive peak may appear smaller on the Raman spectra due to the polarization change during the vibration. The peak which appears on the FT–IR spectrum at 1700–1800 cm^−1^ is due to the asymmetric stretch, which was observed on S=O, whereas the vibration observed at 1045–1060 cm^−1^ is due to S=O being observed as a symmetric stretching vibration.

Raman spectroscopy of compound (**I**) revealed characteristic vibrational modes consistent with its functional groups as summarized in Table 4 ▸. A strong band at 3065 cm^−1^ corresponds to N—H stretching of the protonated amine (–NH_3_^+^) groups, while very strong peaks at 2935 and 2905 cm^−1^ arise from aliphatic C—H stretching. Aromatic ring C=C stretching modes appear at 1615 and 1590 cm^−1^. A medium-intensity band at 1460 cm^−1^ is attributed to C—H bending vibrations. Strong bands in the 1025–990 cm^−1^ region correspond to symmetric and asymmetric S=O stretching of the sulfate anion, and medium-intensity peaks at 790–730 cm^−1^ are assigned to SO_4_^2−^ bending deformations, consistent with typical tetrahedral sulfate vibrational behaviour.

Thermogravimetric analysis (TGA) and dif­ferential scanning calorimetry (DSC) were used to investigate the thermal behaviour of salt (**I**). The TGA data show a gradual decrease in weight from about 200 °C, indicating the onset of thermal decomposition. Although no distinct inflexion is observed below 200 °C, a slight drift in the baseline of the derivative thermogravimetric (DTG) curve may suggest minor desorption of surface-bound solvent or water in that region. This is supported by the DSC trace, which displays a shallow endothermic event in the same range, consistent with solvent loss rather than melting.

**Figure 8:**
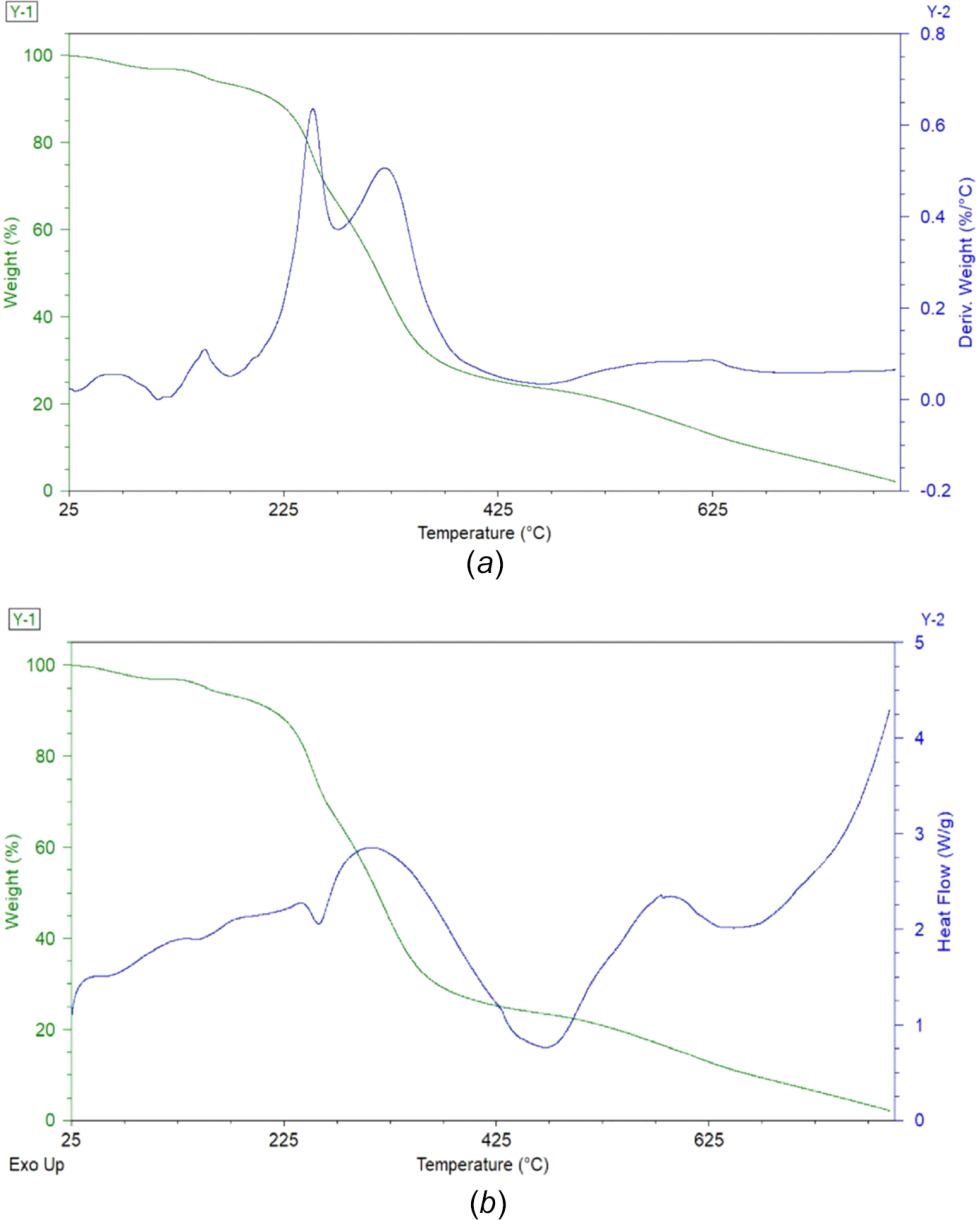
(*a*) TGA–DTG data and (*b*) TGA–DSC data, indicating a mass loss due to the decom­position of the sulfate anion and the 2-hy­droxy-2,3-di­hydro-1*H*-inden-1-aminium cation.

Notably, a well-defined melting endotherm is absent, con­firm­ing that salt (**I**) does not exhibit a simple melting transition under these conditions. With further heating, an exothermic transition is observed at approximately 306.6 °C (579.75 K), which likely marks the beginning of decom­position (possibly involving the loss of the C_18_H_24_N_2_O_2_ fragments). The thermal event observed near 325–350 °C indicates the total decomposition of the sulfate SO_4_^2−^ anion (Brown, 1988 ▸). Fig. 8 ▸ illustrates these thermal events, where the initial weight loss and corresponding endothermic DSC feature collectively emphasize the removal of residual solvent/moisture, with later peaks arising from com­pound degradation rather than phase transitions.

The ^1^H NMR exhibits multiplet signals at δ 2.91 to 2.95 ppm and δ 3.00 to 3.03 ppm due to the aliphatic protons of CH_2_. The OH group produced a single broad peak at δ 5.83 ppm. A doublet was observed because of one CH(OH) proton. The four protons on the indenol aromatic ring were responsible for the multiplet that was observed. At δ 8.50, 8.13 and 7.71, three single peaks were observed due to the NH_3_ protons.

In the ^13^C NMR spectral analysis, the aliphatic region shows the signal due to CH_2_ at δ 35.53 ppm, while the second aliphatic carbon signal due to C—NH_3_ appears at δ 62.44 ppm. The third aliphatic carbon signal is due to CH(OH) at δ 70.17 ppm. A signal due to one carbon at δ 126.77 ppm was observed. Another signal due to the two aromatic carbons of the indenol moiety was observed at δ 128.85 ppm. At δ 129.13 ppm, a signal due to one carbon (–CH) on the aromatic region was observed. A signal due to –C(CH)= was observed at δ 138.62 ppm. A signal due to the carbon in the aromatic ring was observed at δ 141.40 ppm.

## Supplementary Material

Crystal structure: contains datablock(s) I, global. DOI: 10.1107/S2053229625005807/dg3077sup1.cif

Structure factors: contains datablock(s) I. DOI: 10.1107/S2053229625005807/dg3077Isup2.hkl

Supporting information file. DOI: 10.1107/S2053229625005807/dg3077sup3.mol

Supporting information file. DOI: 10.1107/S2053229625005807/dg3077Isup4.cml

CCDC reference: 2324379

## Figures and Tables

**Table 1:** Experimental details

Crystal data	
Chemical formula	2C_9_H_12_NO^+^·SO_4_^2−^
*M*_r_	396.47
Crystal system, space group	Monoclinic, *P*2_1_
Temperature (K)	123
*a*, *b*, *c* (Å)	11.6412 (10), 6.4226 (4), 12.8761 (10)
β (°)	103.791 (4)
*V* (Å^3^)	934.95 (12)
*Z*	2
Radiation type	Mo *K*α
μ (mm^−1^)	0.21
Crystal size (mm)	0.57 × 0.11 × 0.03

Data collection	
Diffractometer	Bruker APEXII CCD
No. of measured, independent and observed [*I* ≥ 2σ(*I*)] reflections	26758, 3458, 2956
*R*_int_	0.098
(sin θ/λ)_max_ (Å^−1^)	0.606

Refinement	
*R*[*F*^2^ > 2σ(*F*^2^)], *wR*(*F*^2^), *S*	0.045, 0.111, 1.04
No. of reflections	3454
No. of parameters	276
No. of restraints	1
H-atom treatment	H atoms treated by a mixture of independent and constrained refinement
Δρ_max_, Δρ_min_ (e Å^−3^)	0.28, −0.42
Absolute structure	Hooft *et al.* (2010 ▸)
Absolute structure parameter	−0.04 (6)

Computer programs: *APEX3* (Bruker, 2016 ▸), *SAINT* (Bruker, 2016 ▸), *SHELXT* (Sheldrick, 2015*a* ▸), *PLATON* (Spek, 2020 ▸), *ORTEP-3 for Windows* (Farrugia, 2012 ▸), *Mercury* (Macrae *et al.*, 2020 ▸), *olex2.refine* (Bourhis *et al.*, 2015 ▸), *SHELXL* (Sheldrick, 2015*b* ▸) and *OLEX2* (Dolomanov *et al.*, 2009 ▸).

**Table 2:** Selected bond angles (°)

O4—S1—O3	110.40 (14)	O6—S1—O3	110.36 (13)
O5—S1—O3	110.40 (13)	O6—S1—O4	108.23 (13)
O5—S1—O4	108.36 (14)	O6—S1—O5	109.02 (13)

**Table 3:** FT–IR spectral data analysis of (**I**)

Group	Wavenumber observed (cm^−1^)	Wavenumber (cm^−1^)	Intensity	Assignment
–NH_2_	2995–2849	3200–3180	Strong	NH_2_ primary amine
–OH	3003–2868	3100–2400	Very strong	OH stretch, very broad band
–NH_2_	1650	1650–1580	Mid-strength	NH_2_ deformation
S=O	1038	1060–1045	Very strong	S=O stretch

**Table 4:** Raman spectral data analysis of (**I**)

Group	Wavenumber observed (cm^−1^)	Wavenumber (cm^−1^)	Intensity	Assignment
–NH_3_^+^	3065	3200 – 3100	Strong	N—H stretch
C—H	2935/2905	3000–2800	Very strong	Aliphatic C—H stretching
C=C	1615/1590	1650–1580	Strong	C=C aromatic ring stretch
C—H	1460	1500–1300	Mid-strength	C—H deformation
S=O	1025–990	1100–980	Strong	S=O symmetric/asymmetric stretching
SO_4_^2−^	790–730	750–600	Mid-strength	SO_4_^2−^ bending deformation
